# Selective reprogramming of regulatory T cells in solid tumors can strongly enhance or inhibit tumor growth

**DOI:** 10.3389/fimmu.2023.1274199

**Published:** 2023-10-20

**Authors:** Rami Alfar, John V. Napoleon, Imrul Shahriar, Richard Finnell, Cole Walchle, Austin Johnson, Philip S. Low

**Affiliations:** ^1^ Department of Chemistry, Purdue Institute for Drug Discovery, Purdue University, West Lafayette, IN, United States; ^2^ Departments of Molecular and Cellular Biology, Molecular and Human Genetics and Medicine, Baylor College of Medicine, Houston, TX, United States

**Keywords:** regulatory T cells, folate receptor-delta, tumor immunosuppression, immunomodulation, immunotherapy of cancer

## Abstract

Folate receptor delta (FRδ) has been used as a biomarker for regulatory T cells (Tregs), because its expression is limited to Tregs and ovum. Although FRδ is unable to bind folate, we have used molecular docking software to identify a folate congener that binds FRδ with high affinity and have exploited this FRδ-specific ligand to target attached drugs (imaging agents, immune activators, and immune suppressors) specifically to Tregs in murine tumor xenografts. Analysis of treated tumors demonstrates that targeting of a Toll-like receptor 7 agonist inhibits Treg expression of FOXP3, PD-1, CTLA4, and HELIOS, resulting in 40-80% reduction in tumor growth and repolarization of other tumor-infiltrating immune cells to more inflammatory phenotypes. Targeting of the immunosuppressive drug dexamethasone, in contrast, promotes enhanced tumor growth and shifts the tumor-infiltrating immune cells to more anti-inflammatory phenotypes. Since Tregs comprise <1% of cells in the tumor masses examined, and since the targeted drugs are not internalized by cancer cells, these data demonstrate that Tregs exert a disproportionately large effect on tumor growth. Because the targeted drug did not bind to Tregs or other immune cells in healthy tissues, the data demonstrate that the immunosuppressive properties of Tregs in tumors can be manipulated without causing systemic toxicities associated with global reprogramming of the immune system.

## Introduction

The human immune system has evolved to protect against pathogens and mutated cells by first mediating their destruction and then facilitating the healing of any damaged tissue (1, 2). Unwarranted activation or delayed termination of the inflammatory phase of an immune response can lead to inflammatory diseases (e.g. rheumatoid arthritis (3), multiple sclerosis (4), Crohn’s disease (5), or psoriasis (6), etc.), while unstimulated inauguration or failed termination of the healing phase of the same response can lead to fibrotic diseases (e.g. pulmonary fibrosis (7), liver cirrhosis (8), chronic kidney disease (9) or cardiac fibrosis (10). Multiple regulatory pathways are therefore operative in virtually all immune cells to prevent emergence of such autoimmune diseases.

Regulatory T cells (Tregs) constitute a major immune cell type involved in maintenance of the balance between pro-inflammatory and anti-inflammatory functions of the immune system (11). Tregs are differentiated from naïve CD4 single positive T cells by IL-2 plus TGFβ and/or T cell receptor engagement, resulting in expression of FOXP3, upregulation of checkpoint receptors (e.g. CTLA4, LAG3, and PD-1), release of immunosuppressive cytokines (i.e. TGFβ, IL-10 and IL-35), scavenging of IL-2, and granzyme-mediated killing of cytotoxic T cells, dendritic cells and NK cells (12). Tregs can also secrete growth factors that stimulate proliferation and survival of cancer cells (12–14). While all of these activities can aid in repair of a wound or pathogen-damaged tissue (15), in the context of a tumor mass they are harmful and probably responsible for the strong correlation between Treg abundance and poor patient survival (16, 17).

Based on the negative impact of Treg infiltration on cancer patient survival, many strategies have been explored to reduce Treg numbers and/or inhibit their functions in cancer patients (14, 18, 19). Cyclophosphamide, EZH2 inhibitors, sorafenib, sunitinib, imatinib, and BET inhibitors, as well as antibodies against CD25, FRδ, GITR, CCR4, CCR8, NRP-1 and CTLA4 (19–24) have been tested for their abilities to reduce Treg immunosuppression. However, none of these strategies has achieved FDA approval either because of inadequate potency or off-target toxicities. Although several creative approaches remain under investigation (23–25), a need still exists to suppress the harmful activities of Tregs in a tumor microenvironment (TME) without compromising their essential functions in healthy tissues.

In the studies below, we exploit a receptor that is almost exclusively expressed on Tregs (i.e. folate receptor delta; FRδ) (26, 27) and that surprisingly becomes functional only upon Treg infiltration into malignant tissues. We then employ molecular docking software to identify a ligand that can bind to this receptor and use this ligand to deliver an attached immune modulating drug specifically into Tregs in cancer tissues. We finally demonstrate that delivery of a TLR7 agonist to Tregs in the TME can reprogram the TME to a less immunosuppressive state, leading to significantly reduced tumor growth and enhanced mouse survival. We also show that targeting of an immunosuppressive steroid to Tregs in the TME can actually enhance tumor growth. Considering the many immunotherapies that could benefit from a more responsive immune microenvironment, this ability to manipulate Tregs to become either more or less immunosuppressive could attract considerable application.

## Materials and methods

### Cell lines

4T1 (Cat# CRL-2539, ATCC) and CT26 (Cat# CRL-2638, ATCC) cells were maintained in complete RPMI (RPMI1640 supplemented with 10% Fetal Bovine Serum (FBS), 1% penicillin-streptomycin (10,000 U/mL stock) and 1% of 200mM L-Glutamine). MB49 (Cat# SCC148, Sigma Aldrich) cell lines were cultured in complete DMEM (DMEM supplemented with 10% FBS, 1% penicillin-streptomycin (10,000 U/mL stock) and 1% of 200mM L-Glutamine Cells). Cells were maintained in 75 cm^2^ tissue culture–treated plastic flasks and used within 10 passages of thawing the cells from frozen stocks.

### Animals

BALB/c and C57BL/6 female mice were purchased from Charles River Laboratories and housed in accordance with Purdue Animal Use and Care Committee guidelines. FRδ knockout mice embryos (Item# 037093-UNC-EMBRYO, MMRRC) were purchased and mice obtained in collaboration with Purdue University’s Transgenic and Genome Editing Facility. FRβ knockout mice were generously provided by Dr. Richard Finnell (Baylor College of Medicine, Houston TX). Mice were first blindly randomized and used at 8–12 weeks of age for tumor implantation studies, and unless otherwise specified, were housed on corn cob bedding, and placed on folate deficient chow (Teklad Envigo) for 2 weeks prior to use in experiments. 4T1 and CT26 cancer cells (1 × 10^5^ cells per mouse) were implanted subcutaneously in BALB/c mice, whereas MB49 cells were similarly implanted in C57BL/6 mice, after which mice were further randomized based on tumor volume. Tumor volume (V) was quantitated using the formula V= LxW^2^/2, where L is the length of the tumor’s longest dimension and W is the width perpendicular to this dimension. Treatments were initiated when tumors reached 50 mm^3^ and harvested at 600–800 mm^3^ for analysis unless otherwise specified.

### Human Treg isolation

Human blood was collected from healthy volunteers (IRB approval number: IRB-2021-1246). Ficoll-Paque separation method was used to obtain PBMCs. Briefly, blood was diluted with 1:1 in 2% FBS in PBS. 50 mL SepMate tubes (Cat# 85450, StemCell Technologies) were prefilled with ~15mL Ficoll (Cat# GE17-5442-02, Sigma Aldrich). Diluted blood was then poured slowly into the tubes, after which the tubes were centrifuged at 12,000 G for 10 minutes. PBMCs were collected and washed twice with cold 2% FBS in PBS. Tregs were isolated from PBMCs using EasySep Human CD4+CD127lowCD25+ Regulatory T Cell Isolation Kit (Cat# 18063, StemCell Technologies) according to the manufacturer’s protocol. Tregs were resuspended in 5% human serum in PBS for downstream applications.

### Murine Treg and effector T cell isolation

For murine Treg isolation, organs and tissues were removed, mechanically disrupted, and digested using Collagenase I (Cat# C0130, Sigma Aldrich) dissolved in complete RPMI (RPMI1640 supplemented with 10% Fetal Bovine Serum (FBS), 1% penicillin–streptomycin (10,000 U/mL stock) and 1% of 200mM L-Glutamine) at a final concentration of 1mg/ml prior to incubation at 37C for one hour with continuous shaking. Digested tissues were resuspended in 1X RBC lysis buffer (Cat# 420301, BioLegend) for 10 minutes, washed and filtered through a 70 um filter to obtain single cell suspensions. Tregs were purified using EasySep mouse CD4+CD25+ Regulatory T Cell Isolation Kit II (Cat# 18783, StemCell Technologies) according to the manufacturer’s protocol, and CD4+CD25- effector T cells were isolated using the same kit. Isolated cells were resuspended in staining buffer for downstream application.

### Flow cytometry analysis

Human Tregs were labeled with antibodies against CD4 (Cat# 317408, BioLegend), CD25 (Cat# 302610, BioLegend) and CD127 (Cat# 351319, BioLegend), and murine Tregs were labeled with antibodies against CD45 (Cat# 103139, BioLegend), CD4 (Cat# 100514, BioLegend), CD25 (Cat# 102049, BioLegend), FRδ (Cat# 125005, BioLegend), and FOXP3 (Cat# 126403, BioLegend). For transcription factors staining, Transcription Factor Staining Buffer Set (Cat #00-5523-00, eBioscience, San Diego, CA) was used according to manufacturer’s protocol. All flow cytometry sample analyses were carried out on Attune NXT analyzer with data analysis performed using FlowJo v10.7.2 analysis software.

### *In vivo* imaging studies

Eight-week-old BALB/c mice were implanted with 4T1 tumors (1 × 10^5^ cells per mouse) with one flank tumor on each animal, and mice were randomized. Once tumors reached a size of approximately 600 mm^3^, mice were injected with 10 nmols of raltitrexed-S0456 either with or without 100-fold excess of competitor (raltitrexed-glucosamine), or 10 nmols of folate-S0456 with or without 100-fold excess competitor (folate-glucosamine). Two hours later, mice and the excised organs were imaged using Spectral Ami Optical Imaging System. The tumors were digested using Mouse Tumor Dissociation Kit (Cat# 130-096-730, Miltenyi Biotech) according to manufacturer’s protocol. Digested tumor cells were resuspended in 1X RBC lysis buffer (Cat# 420301, BioLegend) for 10 minutes, washed and filtered through 70 um filters, and resuspended in 2% FBS in Phosphate Buffered Saline (PBS) to obtain single-cell suspensions. Spleens and other tissues from the same mice were isolated as described previously to obtain single-cell suspensions.

### CFSE proliferation assay

Murine CD4+CD25- effector T cells (Teff) were obtained as described previously. Cells were stained with Carboxyfluorescein succinimidyl ester (CFSE) using CellTrace™ CFSE Cell Proliferation Kit (Cat #C34554, ThermoFisher Scientific). Briefly, cells were suspended at 1 x 10^6^ per milliliter of serum-free PBS, and CFSE was added with agitation at 5uM final concentration. Cells were incubated for 10 minutes at room temperature. CFSE dye was quenched with 3X serum-containing media, which was added with gentle agitation. Quenched media was put on ice for 5 minutes and washed twice. Tregs (either treated or untreated) and Teff cells were cocultured at 1:4 ratio in complete RPMI with or without mouse T-cell activator CD3/CD28 beads (Cat #11456D, ThermoFisher Scientific). After four days, samples were analyzed on flowcytometry to track proliferation of CD4+CD25- Teff cells (28).

### IL-10 and TGF-beta ELISA assay

Tregs and Teff cells were isolated as described previously. Tregs were incubated with or without treatment for 3 hours, then cells were co-cultured at 1:4 ratio of Tregs : Teff cells in complete RPMI. Cells were then incubated with or without mouse T-cell activator CD3/CD28 beads for 48 hours. Subsequently, supernatant was collected and either used directly for ELISA assay or stored at -80 for later analysis.

### Therapeutic studies

BALB/c mice or C57BL/6 (6 to 8-week-old) were implanted with 0.5 × 10^5^ 4T1 tumor (subcutaneously). Treatment was initiated when tumors reached 50 mm^3^. Treatments were carried out daily for 5 days per week. Tumor growth and animal weight change were monitored during the study.

### General experimental information

All reagents were purchased from commercial sources and were used without further purification. All the reagents and chemicals for synthesis were purchased from Sigma-Aldrich (St. Louis). All other cell culture reagents, syringes, and disposable items were purchased from VWR (Chicago, IL). All preparative HPLC was performed with an Agilent 1200 Instrument with a reverse-phase XBridge OBD preparative column (19 × 150 mm, 5 μm) manufactured by Waters (Milford, MA) with UV detection at 254 nm. LC/MS was performed on an Agilent 1220 Infinity LC with a reverse-phase XBridge Shield RP18 column (3.0 × 50 mm, 3.5 μm). The purity of all final compounds was ≥95% as determined by analytical HPLC on a reverse-phase column with the binary system ammonium acetate (20 mM, pH – 7) and acetonitrile as eluent.

### Statistical analysis

Statistical analysis was performed using a Student’s t-test (paired, single-tail analysis) with data represented as mean ± SEM. Prism 7.0 (GraphPad) was used for all statistical analyses and graph generation. Significance is shown as ns, P > 0.05; *, P ≤ 0.05; **, P ≤ 0.01; ***, P ≤ 0.001; ****, P ≤ 0.0001.

## Results

### Identification of a ligand for targeting attached drugs to FRδ on regulatory T cells

**Figure 1:**
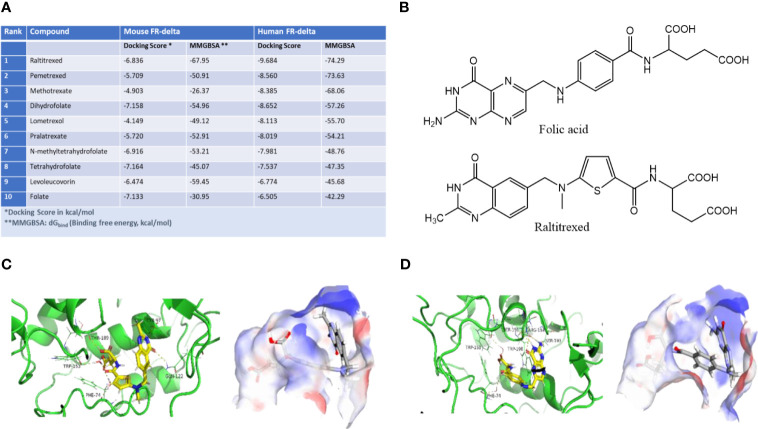
Identification of a high affinity ligand for folate receptor delta (FRδ). **(A)** Predicted relative binding free energies (MMGBSA dGbind) and docking scores (XP GScore) for binding of folic acid analogs to human FRδ (PDB: 5F4Q). **(B)** Structures of folic acid and raltitrexed. **(C, D)** Binding poses of raltitrexed **(C)** and folic acid **(D)** on human FRδ. Ribbon diagrams display the ligand-protein interactions while surface topographies reveal the electrostatic potential maps and binding orientations of both ligands in the FRδ binding cavity. Blue represents the positively charged regions and red represents the negatively charged regions of the binding site. Similar binding poses of raltitrexed and folate to murine FR-delta are shown in **Supplemental Figure S1**.

As noted in the Introduction, folate receptor delta (FRδ) is only expressed on regulatory T cells (Tregs), a small subset of memory CD4+ T cells and ovum, suggesting that it could constitute a receptor for targeting drugs to Tregs. To explore this hypothesis, we searched for a ligand that would bind to FRδ and deliver an attached drug into Tregs, but display little or no affinity for other cell types. Although FRδ exhibited no affinity for folic acid (26) its strong structural homology with other isoforms of the folate receptor suggested that it might bind a folate-like molecule. To test this possibility, we employed molecular docking software (Glide docking tool in the Schroedinger Suite) to identify a molecule with the folic acid scaffold that might bind FRδ. For this purpose, virtual screening of a library of ~3 million commercially available compounds was performed *in silico*, and their binding free energies (MMGBSA dGbind) and docking scores (XP GScore) for both human and mouse FRδ were calculated. As seen from the representative examples in **Figure 1A**, raltitrexed (panel B), a synthetic antifolate that binds to FRα (29) exhibits one of the lowest MM-GBSA binding free energies for both human (-74.29 kcal/mol) and mouse (-67.95 kcal/mol) FRδ, with several other FDA-approved antifolates exhibiting slightly lower predicted binding affinities (see also binding poses of folate and raltitrexed on human and murine FRδ in panels C & D and **Supplemental Figure 1**). Not surprisingly, folic acid displayed the worst predicted binding free energy among the well-known folate analogs, with values of a -42.29 kcal/mol and -30.95 kcal/mol for human and mouse FRδ, respectively. Docking scores, which are generally less reliable predictors of binding constants, also identified raltitrexed as a strong FRδ ligand (**Figure 1A**). Because raltitrexed had already established a good safety profile in humans (30), we elected to explore its possible use for targeting drugs to Tregs further.

### Characterization of the specificity of raltitrexed for FRδ on regulatory T cells

To directly test whether raltitrexed might bind FRδ, we first synthesized a fluorescently labeled raltitrexed conjugate (Ral-S0456; see synthesis in **Figure S2**) and then examined its ability to bind human and murine Tregs isolated from either peripheral blood or other healthy tissues. As shown in **Figures S3A–D**, fluorescent raltitrexed bound neither human nor murine Tregs, nor did similar fluorescent conjugates of either folic acid or 5-methyltetrahydrofolate (synthesis described in ref. 20). That FRδ was indeed expressed on these murine Tregs (**Figure S3D**), however, was confirmed by showing that an anti-FRδ monoclonal antibody strongly labels the Tregs in the same preparations. We therefore concluded that raltitrexed, folate and 5-methyltetrahydrofolate do not bind FRδ on Tregs isolated from peripheral blood or healthy tissues.

**Figure 2:**
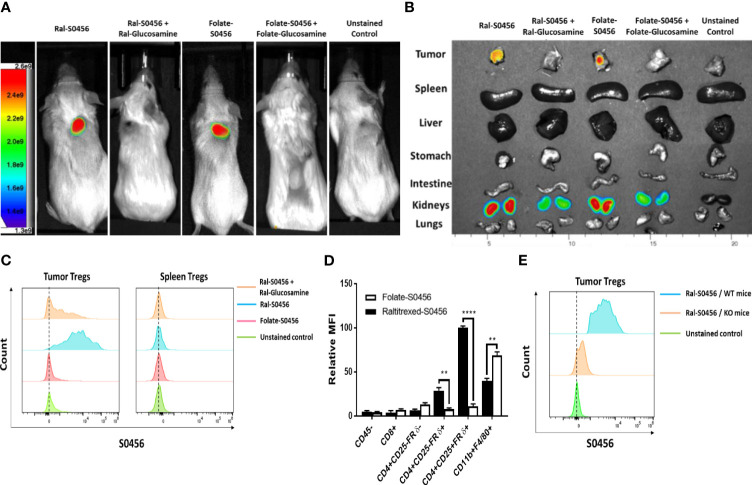
Analysis of the uptake of Raltitrexed-S0456 and Folate-S0456 in 4T1 tumor-bearing mice. Raltitrexed and folic acid were conjugated to the near-infrared fluorescent dye (S0456) as described in **Supplemental Figure S2** and reference (24), and the resulting conjugates were injected intravenously into 4T1 tumor-bearing mice ~2h prior to euthanasia. Whole body **(A)** and organ/tissue **(B)** images were obtained, and tumors were digested using a mouse tumor dissociation kit prior to analysis of cell surface markers by flow cytometry. **(C)** Analysis of CD45+CD4+CD25+FRδ+ Tregs isolated from tumors (left column) but not spleens (right column) of the same mice accumulated Ral-S0456 but not Folate-S0456, and this uptake was competed by Ral-glucosamine. **(D)** Comparison of the uptake of Ral-S0456 and Folate-S0456 by different white blood cell populations in the tumors. **(E)** Flow cytometry data demonstrating that Ral-S0456 accumulates in tumor Tregs of wild type but not FRδ knockout mice, demonstrating that Ral- S0456 uptake is FRδ-receptor mediated. (n=5, data representative of 3 repeated experiments; mean ± SEM; **P < 0.01; ****P < 0.0001).

Because FRβ, an isoform of FR that is only expressed on myeloid cells (31), is known to exist in both a folate binding and nonbinding state, and since it converts from its nonbinding to folate binding state following macrophage infiltration into solid tumors (or sites of inflammation) (32–34), we wondered whether FRδ might similarly convert from a nonbinding to binding state following Treg migration into a tumor mass. To test this hypothesis, we implanted folate receptor negative 4T1 murine breast cancer cells into BALB/c mice and injected the tumor-bearing mice intravenously with the same fluorescent folate-S0456 or Ral-S0456 conjugate employed above (**Figure S2**). As shown in **Figures 2A, B**, both fluorescent conjugates labeled tumors and kidneys (i.e., the site of excretion) but no other tissues, suggesting that the tumor microenvironment must induce an active binding site for both folate and raltitrexed. Moreover, because tumor uptake of the two targeted conjugates could be blocked by co-injection of 100-fold excess nonfluorescent conjugate (i.e., folate- or Ral-glucosamine conjugate), we concluded that their tumor accumulation must be receptor-mediated (see both panels A & B).

In order to identify which cells in the tumor masses took up the fluorescent conjugates, flow cytometry analysis was performed on cells obtained by dissociating the above tumors into single cell suspensions. As shown in **Figure 2C**, CD45+CD4+CD25+FRδ+ cells (i.e., Tregs) specifically captured the Ral-S0456 in a manner that was readily competed with excess Ral-glucosamine, i.e. confirming that the tumor Tregs could indeed bind raltitrexed. Analogous studies of tumor cells isolated from folate-S0456 injected mice similarly confirmed that Tregs did not bind the folate-targeted dye (compare red and blue histograms), i.e., consistent with previous reports that Tregs do not bind folic acid (26). More importantly, CD45+CD4+CD25+FRδ+ cells (Tregs) from spleens of Ral-S0456 injected mice remained nonfluorescent (**Figure 2C**), confirming that FRδ on Tregs in healthy tissues persists in its nonbinding state. Considered together with data demonstrating that ~80% of the CD45+CD4+CD25+FRδ+ cells are indeed FOXP3+ (**Figure S4**), we concluded that raltitrexed does indeed bind Tregs in tumors and that it can be exploited to target attached drugs to Tregs in tumors without delivering the same drugs to nonbinding Tregs in normal tissues.

To more thoroughly characterize the specificities of raltitrexed and folate for the various cells in tumor masses, we next examined the uptake of fluorescent conjugates of both raltitrexed and folate by other cells in the tumor cell suspensions. As shown in **Figures 2D**, **S5A**, CD45- cells were all nonfluorescent, confirming that 4T1 cancer cells, fibroblasts, and endothelial cells etc. do not bind the targeted dyes. Moreover, as expected, all folate receptor negative white cells (CD45+) also remained nonfluorescent (**Figures S5B**, **C**); i.e., concordant with their lack of folate receptors. In contrast, folate-S0456 was aggressively captured by CD45+CD11b+F4/80+FRβ+ cells (tumor-associated macrophages and myeloid derived suppressor cells) (**Figure S5D**), but not by CD45+CD4+CD25+FRδ+ Tregs, whereas Ral-S0456 was primarily accumulated by FRδ+ Tregs and to a lesser extent by CD4+CD25-FRδ+ cells (memory T cells, **Figure S5C**) and CD45+CD11b+F4/80+FRβ+ myeloid cells. This higher uptake of folate-S0456 than Ral-S0456 by FRβ+ tumor-associated macrophages and myeloid derived suppressors cells was consistent with the relative affinities of folate and raltitrexed for FRβ.

To more firmly establish that uptake of Ral-S0456 by Tregs is mediated by FRδ, we next produced a FRδ knockout mouse on a C57BL/6 background and repeated the above *in vivo* labeling studies using a syngeneic tumor (MB49) that grows in C57BL/6 mice. As shown in **Figure 2E**, uptake of Ral-S0456 by tumor-derived Tregs was largely absent in the FRδ knockout mice, confirming that FRδ is responsible for the vast majority of Ral-S0456 accumulation by Tregs. Collectively, the data also established that tumor- but not spleen-associated Tregs bind and internalize raltitrexed- but not folate-dye conjugates via a mechanism that requires a functional FRδ generated in the tumor milieu.

### Identification of an immune modulator for reversing the immunosuppressive activities of Tregs

**Figure 3:**
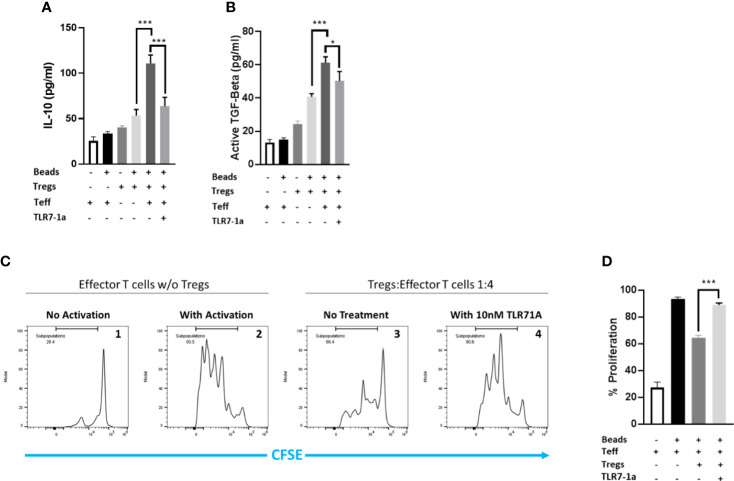
TLR7-1a inhibits the immunosuppressive activities of murine Tregs *in vitro*. Murine Tregs were pre-incubated in culture media either containing or lacking 10 nM TLR7-1a for 3 hours. Tregs were then added to cultures of effector T cells (1:4; Treg:_Teff_) and allowed to incubate for 48 hours, after which culture media was collected and analyzed for **(A)** IL-10 or **(B)** TGFB. **(C)** Murine effector T cells were pre-labeled with carboxyfluorescein succinimidyl ester prior to incubation either without (panel 1) or with (panels 2-4) anti-CD3/CD28 beads to stimulate their proliferation. Tregs that were either not pretreated (panel 3) or pretreated (panel 4) with 10 nM TLR7-1a were co-incubated in the cell cultures and cells were allowed to proliferate for four days. Expansion of the prelabeled effector T cells was then monitored by flow cytometry by measuring the decrease in effector T cell fluorescence as the internalized carboxyfluorescein was diluted with each cell division. **(D)** Cell divisions were quantitated by calculating the area under the curve to the left of the non-dividing T-cell peak. (n=5, data representative of 3 repeated experiments; mean ± SEM; *P < 0.05; ***P < 0.001).

With the ability of raltitrexed to deliver an attached molecule selectively to tumor-infiltrating Tregs established, we next hypothesized that a raltitrexed-linked drug might be exploited to reduce Treg immunosuppression in solid tumors without perturbing immune functions in healthy tissues. To test this hypothesis, we first compared the abilities of several immune modulators to lower production of the immunosuppressive cytokine, IL-10, by tumor-derived Tregs. As shown in **Figure S6**, a Toll-like receptor 7 agonist (TLR7-1a) (32, 35) was found to suppress IL-10 biosynthesis better than inhibitors of HDAC8, EZH2, or Dyrk1a (see also **Figure 3A**). Importantly, the same TLR7 agonist was also successful in inhibiting release of the immunosuppressive cytokine, TGFβ (**Figure 3B**) and in reversing the ability of Tregs to block proliferation of effector T cells (compare panels 1-4 of **Figure 3C**). The proliferation was quantitated by calculating the area under the curve of the dividing cells to the left of the non-dividing T-cell peak, as illustrated in the bar graph (**Figure 3D**). Based on these observations, we concluded that TLR7-1a would constitute an excellent candidate for assessing the capability of raltitrexed to deliver an immune modulating drug specifically to Tregs *in vivo*.

### Evaluation of the ability of raltitrexed-targeted immune modulators to reprogram tumor-infiltrating Tregs *in vivo*

**Figure 4:**
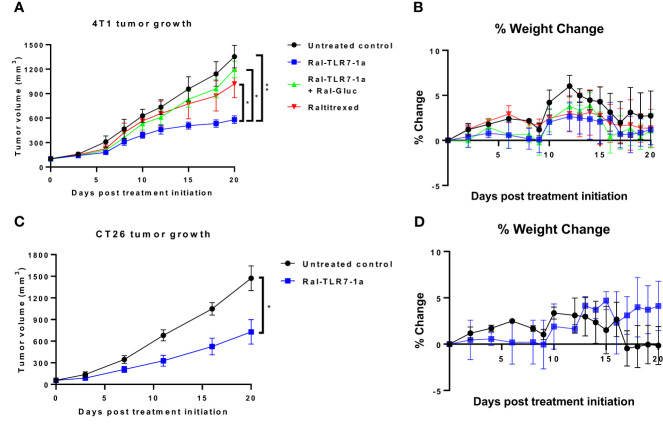
Effect of Ral-TLR7-1a on tumor growth and body weight in murine breast 4T1 and murine colorectal CT26 subcutaneous tumors. BALB/c mice were implanted with syngeneic 4T1 cancer cells and were treated with either buffer (untreated control), raltitrexed, Ral-TLR7-1a or Ral-TLR7-1a with Ral-Gluc, where tumor volumes **(A)** and body weights **(B)** were assessed every other day for 20 days. Additionally, BALB/c mice were implanted with syngeneic CT26 cancer cells and were treated with either buffer (untreated control) or Ral-TLR7-1, where tumor volumes **(C)** and body weights **(D)** were assessed every other day for 20 days. (n=5 mice/group, data representative of 2 repeated experiments; mean ± SEM; ns, P > 0.05; *P ≤ 0.05; **P ≤ 0.01).

To explore the ability of Ral-TLR7-1a to modulate the immunosuppressive properties of Tregs in solid tumors, raltitrexed was linked via a self-immolative spacer to TLR7-1a (see synthesis in **Figure S7**) such that upon internalization of the conjugate (hereafter referred to as Ral-TLR7-1a), the consequent reduction of the disulfide bond in the spacer would trigger release of an unmodified TLR7-1a into the Treg. To quantitate the potency of the targeted Ral-TLR7-1a, immune competent mice with 4T1 syngeneic tumors were treated via tail vein injection with 10 nmoles Ral-TLR7-1a and examined for changes in both tumor growth and body weight. As shown in **Figure 4A** (left panel), Ral-TLR7-1a reduced tumor growth by ~60% relative to untreated controls. Free raltitrexed, in contrast, inhibited tumor growth by only 20%, suggesting that the majority of growth inhibition by Ral-TLR7-1a was contributed by the TLR7-1a (none of these treatments caused significant mice body weight changes, as illustrated in **Figure 4B**) (36). That this growth inhibition was receptor-mediated could then be demonstrated by showing that blockade of FRδ with 100-fold excess Ral-glucosamine reversed the inhibition almost quantitatively (green curve). Moreover, the observation that none of the treated animals lost weight during the three-week therapy suggests that Ral-TLR7-1a was not inhibiting the essential functions of Tregs in healthy tissues and was therefore not toxic to the animals. And since analogous results were obtained when mice implanted with CT26 syngeneic colon cancer tumors were similarly treated (**Figures 4C, D**), we conclude that Ral-TLR7-1a inhibition of tumor growth is likely a property of most Treg-containing tumors.

### Evaluation of the mechanism of Ral-TLR7-1a modulation of tumor proliferation

**Figure 5:**
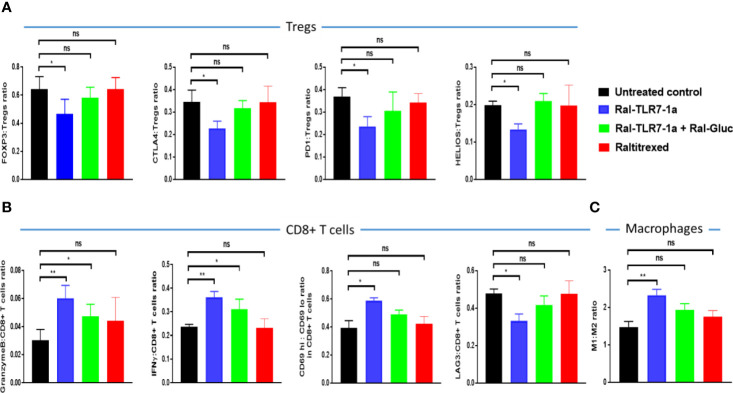
Evaluation of the effect of Ral-TLR7-1a on phenotypic markers of tumor-infiltrating Tregs **(A)**, CD8+ T cells **(B)**, and tumor-associated macrophages **(C)**. Following euthanasia, tumors were excised and dissociated using mouse Tumor Dissociation Kit prior to analysis of the component cells by flow cytometry. The relevant phenotypic markers are listed on the y axis and the treatment regimen is indicated by the color of the bar. (n=5, data representative of 2 repeated experiments; mean ± SEM; ns, P > 0.05; *P < 0.05; **P < 0.01).

In order to define the mechanism by which Ral-TLR7-1a inhibits tumor growth in greater detail, we dissociated the tumor masses into single cell suspensions and examined their diagnostic markers by flow cytometry. As shown in **Figure 5A**, levels of FOXP3, CTLA4, PD1, and HELIOS on Tregs all decreased upon treatment with Ral-TLR7-1a, suggesting that the Tregs were shifted towards a less immunosuppressive state (37, 38). Consistent with the known engagement of other immune cells by Tregs, activation markers on CD8+ T cells (i.e. granzyme B, IFNγ, and CD69) increased, while a marker of T cell exhaustion (Lag3) decreased (**Figure 5B**), i.e. demonstrating that mitigation of the immunosuppressive properties of Tregs allows effector T cells to become more cytotoxic and less exhausted. Not surprisingly, the CD86 to CD206 ratio on tumor-associated macrophages and myeloid-derived suppressor cells [i.e. often considered a measure of M1:M2 ratio (32, 39)] also increased (**Figure 5C**), further suggesting that the targeted reprogramming of Tregs also impacts tumor-infiltrating myeloid cells. Finally, and perhaps most importantly, analysis of the same phenotypic markers on Tregs from spleens of the same mice revealed no change in any of the diagnostic markers, consistent with the absence of raltitrexed binding sites on splenic Tregs and supportive of the hypothesis that tumor-derived Tregs can be manipulated without altering the properties of Tregs systemically (representative data shown in **Figure S8**).

**Figure 6:**
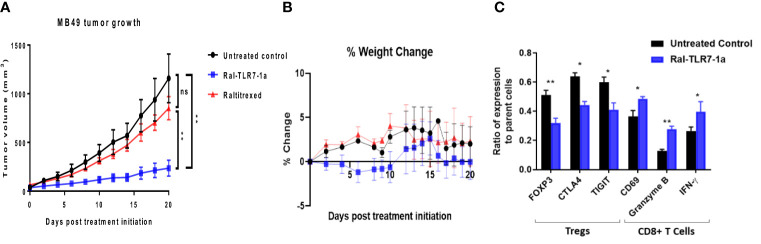
Impact of Ral-TLR7-1a on tumor properties in FRβ knockout mice. FRβ knockout mice on a C57BL/6 background were implanted with syngeneic MB49 cancer cells and treated with either buffer (untreated control), raltitrexed or Ral-TLR7-1a as described in the Methods. Tumor volumes **(A)** and body weights **(B)** were then assessed every other day for 20 days. Following euthanasia, tumors were excised and dissociated as described in Methods prior to analysis of component cells by flow cytometry. Relevant phenotypic markers **(C)** for Tregs (FOXP3, CTLA4 and TIGIT) and CD8+ T cells (CD69, Granzyme B, and IFNγ) were then analyzed in both untreated controls (black bars) and Ral-TLR7-1a treated mice (blue bars) (n=5 mice/group, data representative of 2 repeated experiments; mean ± SEM; ns, P > 0.05; *P < 0.05; **P < 0.01).

It should also be noted that raltitrexed displayed affinity, albeit much weaker, for FRβ on tumor-associated macrophages (**Figure 2D**), suggesting that part of the impact of Ral-TLR7-1a on tumor growth could have derived from its effect on FRβ+ tumor-associated macrophages. Therefore, to obtain a more accurate estimate of the impact of Ral-TLR7-1a solely on Tregs, we repeated the above studies using mice in which we knocked out the gene for FRβ. As shown in **Figure 6**, Ral-TLR7-1a was surprisingly even more effective in suppressing tumor growth in the FRβ knockout mice than in wildtype mice, reducing tumor growth by >80% (compare **Figures 4A**, **6A**). Whether this enhanced potency was a consequence of the specific syngeneic tumor model (i.e., MB49 versus 4T1) or the switch from balb/c to C57Bl/6 mice was not investigated, but the data clearly confirmed that Ral-TLR7-1a mediated reprogramming of tumor Tregs exerts a profound effect on tumor growth even in the absence of any contribution of the drug to suppression of tumor-infiltrating myeloid cells. Mechanistically, the data in panel C also demonstrate that the effect of Ral-TLR7-1a on the MB49 tumor microenvironment is not limited to Tregs; i.e. by reversing the immunosuppressive activities of the Tregs, the anti-inflammatory properties of other immune cells in the 4T1 tumor microenvironment are also improved.

### Stimulation of tumor growth via augmentation of Treg immunosuppression

**Figure 7:**
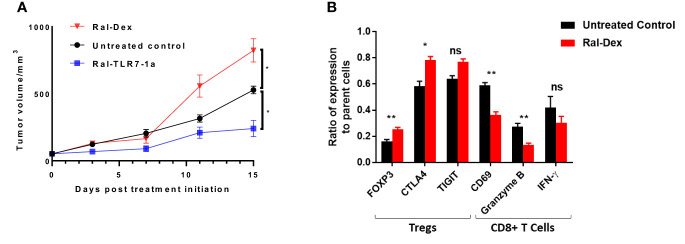
Impact of Raltitrexed-dexamethasone on 4T1 tumor growth in BALB/c mice. BALB/c mice were implanted with syngeneic 4T1 cancer cells and treated with either Ral-Dex, buffer (untreated control), or Ral-TLR7-1a, as described in the Methods. **(A)** Tumor volumes and body weights were then assessed every other day for 20 days. **(B)** Following euthanasia, tumors were excised and dissociated using mouse Tumor Dissociation Kit prior to analysis of the component cells by flow cytometry. Relevant phenotypic markers for Tregs (FOXP3, CTLA4 and TIGIT) and CD8+ T cells (CD69, Granzyme B, and IFNy) were then analyzed in both untreated controls (black bars) and Ral-Dex treated mice (red bars) (n=5 mice/group, data representative of 2 repeated experiments, mean ± SEM; P > 0.05; *P < 0.05; **P < 0.01).

Finally, although the initial purpose of this study was to determine whether tumor-associated Tregs could be manipulated to adopt a less immunosuppressive state, the question naturally arose whether one could also induce Tregs to acquire a more immunosuppressive phenotype. To explore this possibility, we synthesized a dexamethasone conjugate of raltitrexed (Ral-Dex; see **Figure S9** for structures and synthetic schemes) and examined its impact on tumor growth. As shown in **Figure 7A**, targeted dexamethasone actually enhanced tumor growth relative to untreated controls. Moreover, as revealed in **Figure 7B**, this augmentation of growth was predictably accompanied by an opposite shift in phenotypic markers on the other tumor resident immune cells. Taken together, these data suggest that the immunosuppressive potential of tumor-infiltrating Tregs was not maximal in the untreated tumors and that their immunosuppressive functions could in fact be further enhanced by targeted stimulation with an immunosuppressive steroid.

## Discussion

Quantitation of the impact of Tregs on tumor growth has been difficult in the past due to the fact that mice lacking Tregs die within 3-4 weeks of birth and methods to systemically deplete FOXP3+ cells from mature mice have led to serious autoimmune disorders (40–42). Although several researchers have shown that anti-tumor immunity can be potentiated by depletion of Tregs in tumors (43), the unavoidable concurrent reduction of their numbers in healthy tissues has raised questions regarding whether such treatments can be repeatedly administered in humans without causing toxicity (44, 45). The strength of our approach has derived from the unexpected finding that FRδ on peripheral blood and healthy tissue Tregs does not bind raltitrexed (or any other folate congener tested), whereas FRδ on tumor resident Tregs does. With this fortuitous distinction, it became possible to deliver a potent TLR7 agonist to tumor resident Tregs without altering their properties elsewhere, leading to a ~40-80% reduction in tumor growth without inducing obvious systemic toxicities. When considered together with the observations that Tregs comprised <1% of total cells in the tumor masses examined and that tumor-associated macrophages and CD8+ effector T cells are simultaneously repolarized to more inflammatory phenotypes, presumably as a consequence of Treg repolarization, these data argue that Tregs make a disproportionately large contribution to immunosuppression in the tumor TME. This remarkably large impact also suggests that future efforts aimed at augmenting the potencies of checkpoint inhibitors, CAR T cell therapies, bispecific T cell engagers, and adoptive T cell therapies could benefit significantly from co-administration of a Treg-targeted immune stimulant to help prepare the TME for an immunotherapy.

It was surprising to find that FRδ, like FRβ, exists in both a binding and nonbinding state, where the binding state somehow emerges in the tumor microenvironment, but cannot be detected in healthy tissues or peripheral blood (32). Although considerable effort was devoted to identifying the mechanism underpinning this tumor-specific FRδ activation, no obvious post-translational modification could be detected and its activation to a binding state could only be replicated by prolonged incubation of the Tregs in conditioned medium from cancer cell cultures. However, unlike FRβ the binding state of FRδ did not recognize folic acid, but instead associated only with an unnatural antifolate; i.e., raltitrexed. While this unusual ligand specificity may seem strange at first, it be noted that the function of FRδ in oocytes [ie. the other major cell type known to express FRδ (46, 47)] does not involve binding folate, but instead consists of facilitating a stable interaction between an egg and a sperm (26). Thus, FRδ knockout mice are infertile because the Izumo protein on a sperm cannot bind FRδ (a.k.a. Juno, IzumoR1, or FolR4) in the knockout mice on an oocyte (48, 49). Just recently it has been shown that the same FRδ-Izumo interaction facilitates formation of an immunological synapse between Treg and γδT cells, thereby enabling Tregs to suppress γδT cell function (50). Whether this interaction is dependent on conversion of FRδ from a nonbinding to binding state has not been explored, but FRδ activation will clearly be useful for control of immunosuppression in the TME, since it enables selective delivery of immune stimulating drugs to tumor infiltrating Tregs without altering immune cell properties in healthy tissues (51, 52).

The remarkable augmentation of tumor growth by the raltitrexed-dexamethasone conjugate demonstrates that the immunosuppressive capacity of tumor-associated Tregs was not maximized in the tumors examined. While enhancement of tumor growth is unlikely to find application in human medicine, the observation that the immunosuppressive potential of Tregs can be further increased raises the possibility that a raltitrexed-dexamethasone conjugate could prove useful in treating autoimmune diseases such as rheumatoid arthritis, multiple sclerosis, Crohn’s disease or type 1 diabetes, where enhanced immunosuppression might be warranted. Thus, data from other laboratories have clearly established that dysfunctions or insufficient numbers of Tregs are often observed in patients with autoimmune disorders (53–55) and that infusion of additional Tregs can often suppress the inflammation associated with an autoimmune disease (56–58). It will be important in the future to explore whether FRδ on Tregs in autoimmune lesions acquires raltitrexed binding ability, and if a raltitrexed-dexamethasone conjugate can be safely employed to suppress an over-active immune system in patients with an inflammatory disorder such as rheumatoid arthritis, multiple sclerosis or type 1 diabetes

Finally, although a Ral-TLR7-1a caused no overt toxicities in mice, concurrent expression of FRδ on the both the ovum and a subset of memory T cells suggests that future studies must also examine whether these interactions might be deleterious, perhaps interfering with conception or altering immune memory. Thus, if FRδ were to become functional in either cell type and if the same cell type were to also express TLR7, significant changes in the cell’s properties would be expected. Moreover, if functional activation of FRδ were to occur on Tregs under other conditions, the effects of reprogramming the Tregs under such conditions would also require further scrutiny. Nevertheless, the limited expression of FRδ on only three minor cell types, along with the further absence of functional activation under normal conditions in healthy tissues suggests that activation of FRδ in the TME should be exploited for manipulation of this important barrier to the treatment of many cancers.

## Data availability statement

The original contributions presented in the study are included in the article/**Supplementary Material**. Further inquiries can be directed to the corresponding author.

## Ethics statement

Ethical approval was not required for the studies on humans in accordance with the local legislation and institutional requirements because only commercially available established cell lines were used. The animal study was approved by The Purdue Institutional Animal Care and Use Committee (IACUC). The study was conducted in accordance with the local legislation and institutional requirements.

## Author contributions

RA: Data curation, Formal Analysis, Investigation, Methodology, Software, Validation, Writing – original draft, Writing – review & editing. JN: Investigation, Writing – review & editing. IS: Methodology, Software, Writing – review & editing. RF: Investigation, Writing – review & editing. CW: Investigation, Writing – review & editing. AJ: Investigation, Writing – review & editing. PL: Conceptualization, Data curation, Funding acquisition, Project administration, Resources, Supervision, Writing – original draft, Writing – review & editing.
