# Supplementary material for: Selective reprogramming of regulatory T cells in solid tumors can strongly enhance or inhibit tumor growth
Source: Front Immunol. 2023 Oct 20;14:1274199. doi: 10.3389/fimmu.2023.1274199 (PMC10623129; doi:10.3389/fimmu.2023.1274199)

Supplemental Figure 1

A

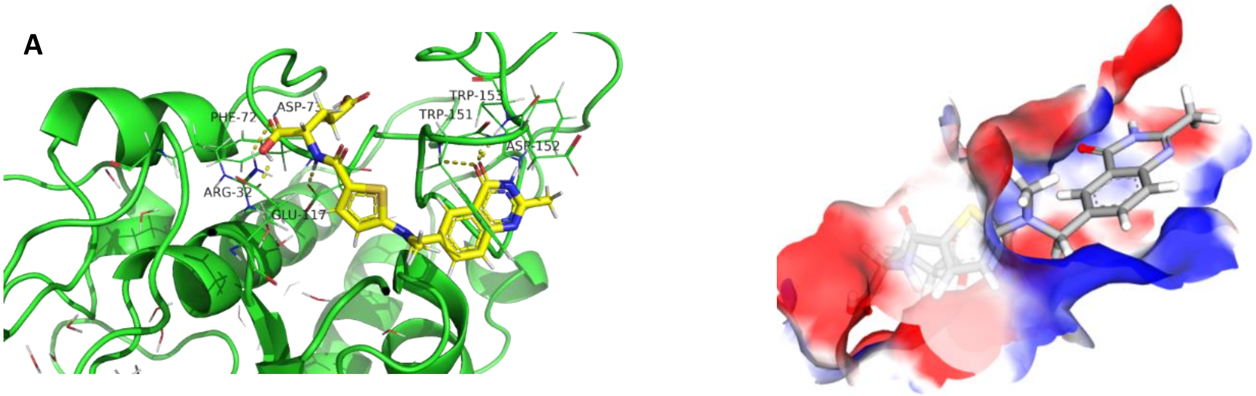

B

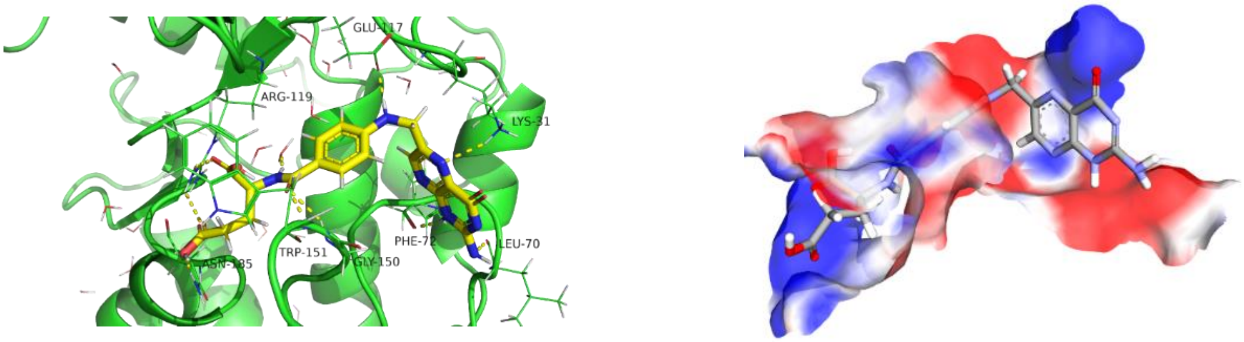

Supplemental Figure 1. Binding poses of A. Raltitrexed on murine FRδ and B. Folate on murine FRδ (PDB:5JYJ)

Supplemental Figure 2

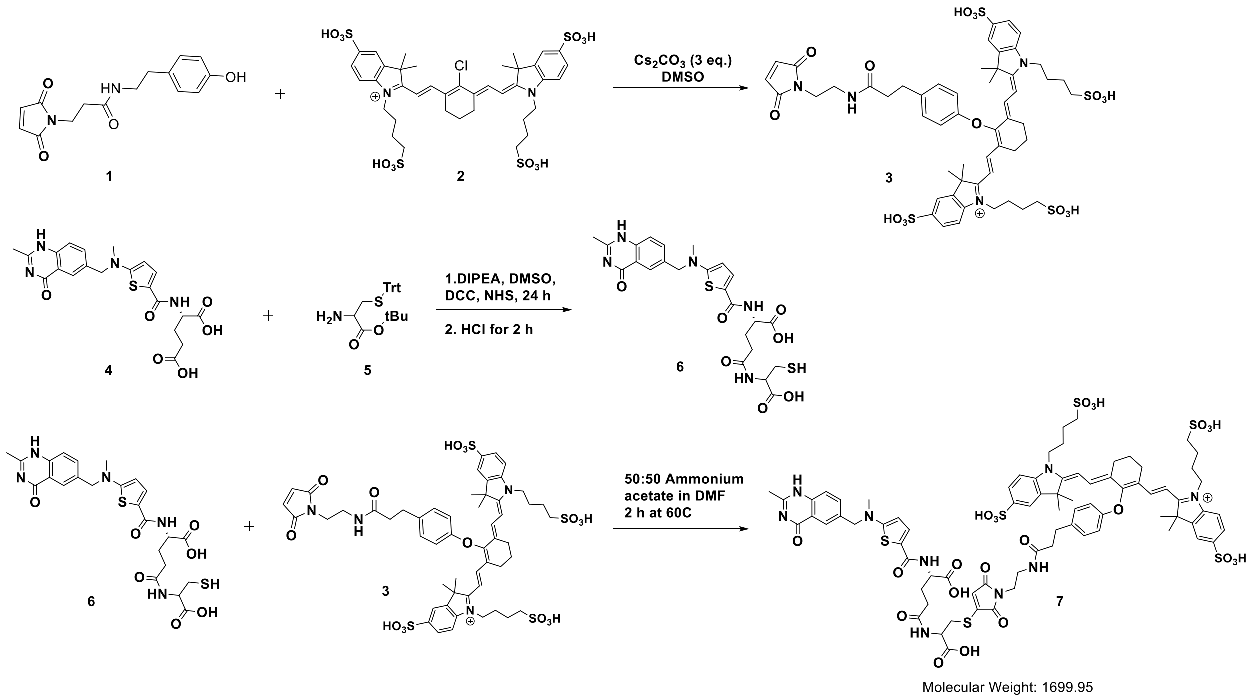

Supplemental Figure 2. Synthetic scheme for the preparation of raltitrexed-S0456 (7) conjugate.

Synthesis of compound 3: To a stirred solution of 3-(2,5-dioxo-2,5-dihydro-1H-pyrrol-1-yl)-N-(4-hydroxyphenethyl)propanamide **1** (0.9 mmol) in DMSO (15 mL), Cs_2CO_3 (2.7 mmol) was added under inert atmosphere. After stirring for five minutes, S0456 intermediate **2** (0.75 mmol) was added to the reaction mixture and stirred for additional 4 h. When the starting materials were completely consumed as determined by LCMS, the reaction mixture was purified by UHPLC using acetonitrile and water mobile phase to get product **3** as a green solid (35%).

Synthesis of compound 6: To a stirred solution of raltitrexed (0.1 mmol) in DMSO (400 μl), DIPEA (50 μl) was added under inert atmosphere. After stirring for 5 minutes, DCC (0.12 mmol) and N-hydroxysuccinimide (0.12 mmol) were added to the reaction mixture and allowed to stir for another 30 minutes. Meanwhile in a separate flask, H-Cys(Trt)-O^{t}Bu.HCl (0.2 mmol) was dissolved in DMSO (400 μl) and DIPEA (50 μl). After stirring for 30 minutes, the cysteine reaction mixture was added to the activated raltitrexed solution and stirred for another 24 h. The progress of the reactions was monitored by LC-MS. Once the starting materials were completely consumed, the crude reaction mixture was purified by UHPLC using 5-95% water and ACN mobile phase system. The combined purified fractions were lyophilized to get the product as a colorless solid (55% yield). To remove the trityl and t-butyl groups, the lyophilized compound was dissolved in excess HCl and stirred for 1 h. After the starting material was completely consumed, it was evaporated to dryness under reduced pressure to get compound **6** as a yellow oil which was used for next step without further purification.

Synthesis of compound 7: To a stirred solution of compound **6** in 1:1 mixture of aqueous ammonium acetate (pH -5) and DMF, S0456-maleimide compound **3** was added and stirred for 2 h at 60 ^{o}C. The crude reaction mixture was purified by UHPLC using 5-95% water and ACN mobile phase system. The combined purified fractions were lyophilized to get raltitrexed-S0456 conjugate (**7**) as a green color solid (Yield - 28%). The product is confirmed by LCMS. (M.Wt. 1699.95 g/mol).

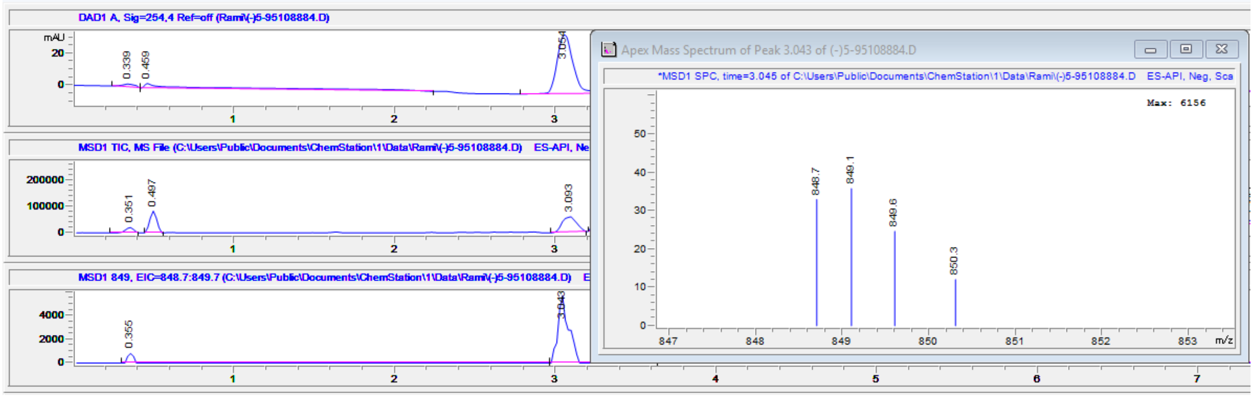

Supplemental Figure 3

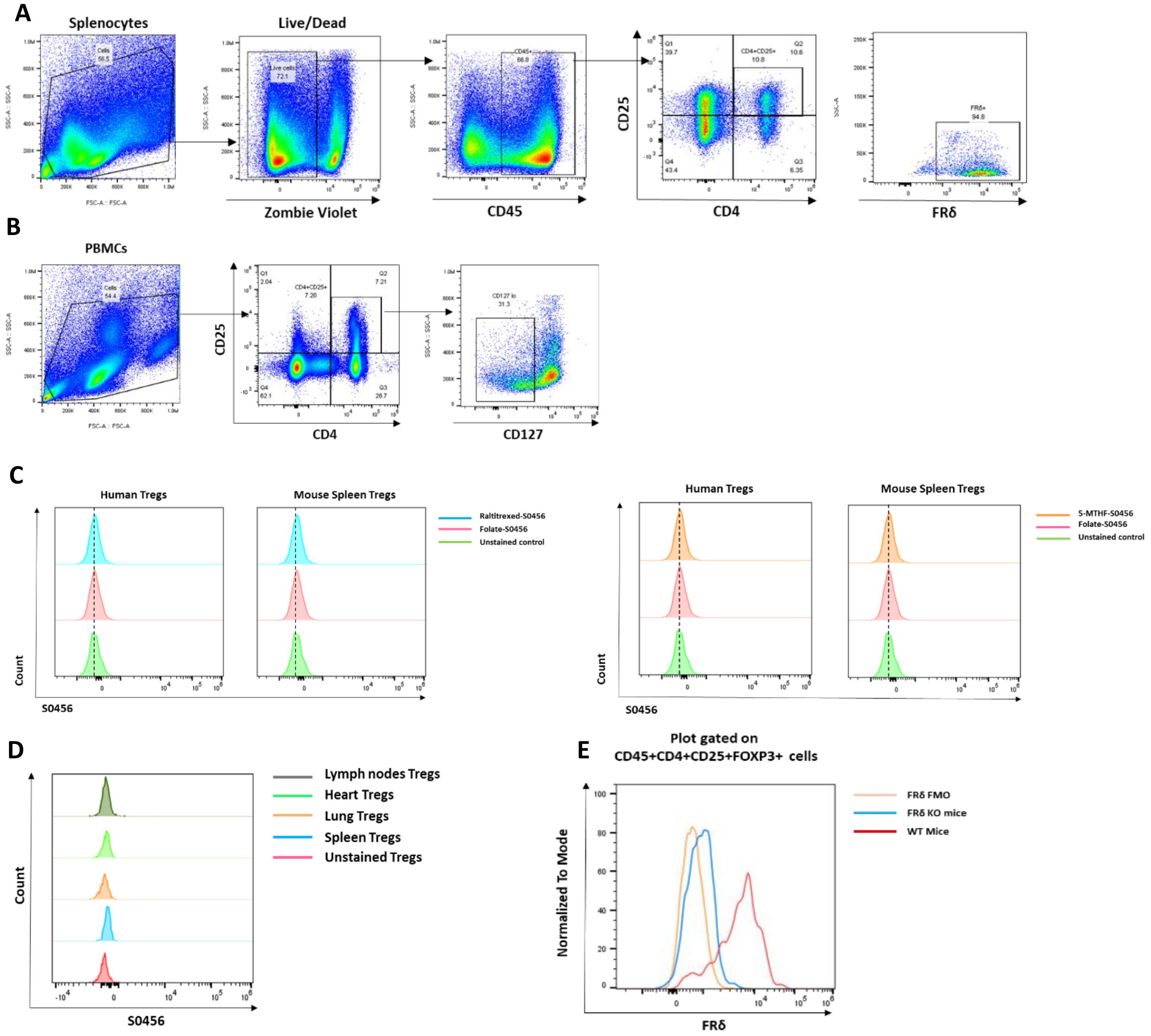

Supplemental Figure 3. Characterization of raltitrexed-S0456 and folate-S0456 binding to Tregs isolated from spleens, peripheral blood, and other organs from healthy mice. A. Gating strategy for characterization of murine spleen CD45+CD4+CD25+FRδ+ Tregs. **B.** Gating strategy for characterization of human CD4+CD25+CD127$_{lo}$/- Tregs. **C.** Flow cytometry histograms showing that neither folate, raltitrexed, nor 5-methyltetrahydrofolate (5-MTHF) bind to human peripheral blood Tregs or murine spleen Tregs. **D.** Flow cytometry histograms showing that Ral-S0456 also does not bind to Tregs isolated from lymph nodes, hearts, lungs, or spleens of healthy mice. Attempts were also made to characterize Tregs from mouse stomachs, livers, kidneys and intestines, but insufficient numbers could be harvested to characterize their binding of Ral-

S0456. **E.** Flow cytometry histograms confirming that a rat anti-mouse FRδ monoclonal antibody binds Tregs on spleen cells from wild type mice but not on Tregs from FRδ KO mice.

Supplemental Figure 4

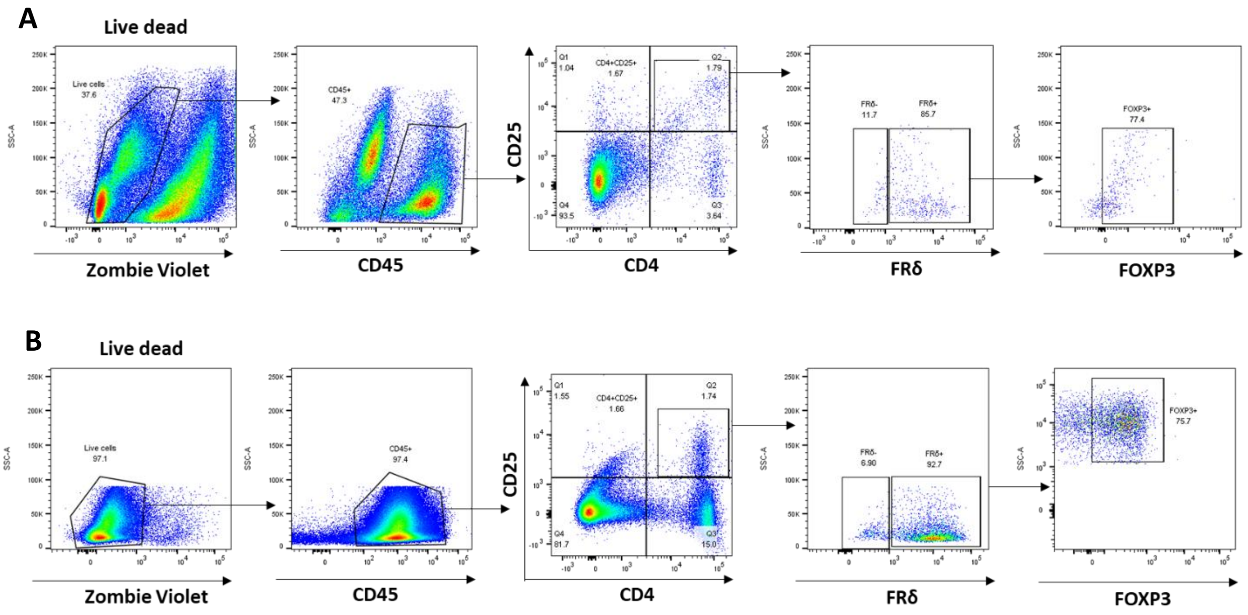

Supplemental Figure 4. Flow cytometry dot plots showing high co-expression of FRδ and FOXP3 on murine tumor and spleen cells. A. Gating strategy for assessment of FOXP3 expression in FRδ positive murine CD45+CD4+CD25+ Tregs isolated from 4T1 tumors. **B.** Gating strategy for assessment of FOXP3 in FRδ positive murine CD45+CD4+CD25+ Tregs isolated from spleens.

Supplemental Figure 5

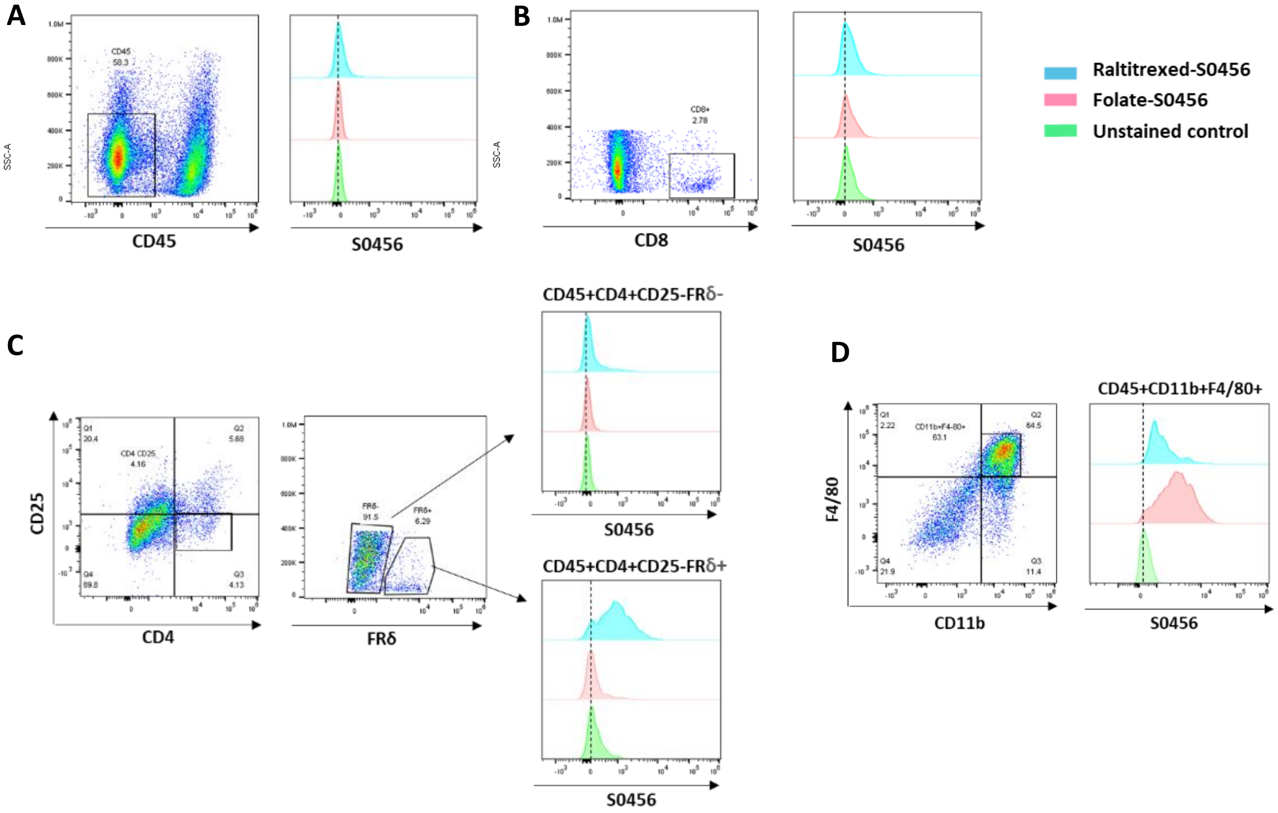

Supplemental Figure 5. Flow cytometry analysis of the uptake of raltitrexed-S0456 and folate-S0456 in different tumor cell types. A. CD45- cells show no binding of either conjugate. **B.** CD45+CD8+ cytotoxic T cells show no binding of either conjugate. **C.** CD45+CD4+CD25-FRδ- cells show no binding of either conjugate, whereas CD45+CD4+CD25-FRδ+ memory T cells show uptake of Ral-S0456 but not folate-S0456. **D.** CD45+CD11b+F4/80 macrophages show significant uptake of folate-S0456 and some uptake of Ral-S0456.

Supplemental Figure 6

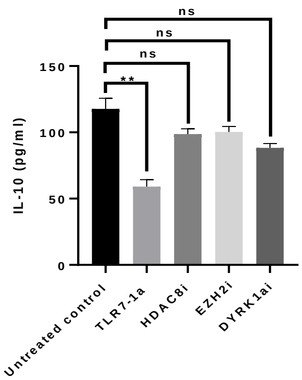

Supplemental Figure 6. Comparison of the abilities of 10 nM TLR7-1a, HDAC8 inhibitor, EZH2 inhibitor and DYRK1a inhibitor to suppress IL-10 production by murine Tregs. Tregs were pre-treated with 10 nM of the indicated compound for 3 hours prior to co-culturing with CD4+CD25- effector T cells for 48 hours in the absence of drug, after which the supernatant was collected and IL-10 was analyzed by ELISA. (HDAC8i: Histone Deacetylase 8 Inhibitor; EZH2i: Enhancer of Zeste Homolog 2 Inhibitor; DYRK1ai: Dual-specificity Tyrosine Phosphorylation-regulated Kinase 1a Inhibitor)

Supplemental Figure 7

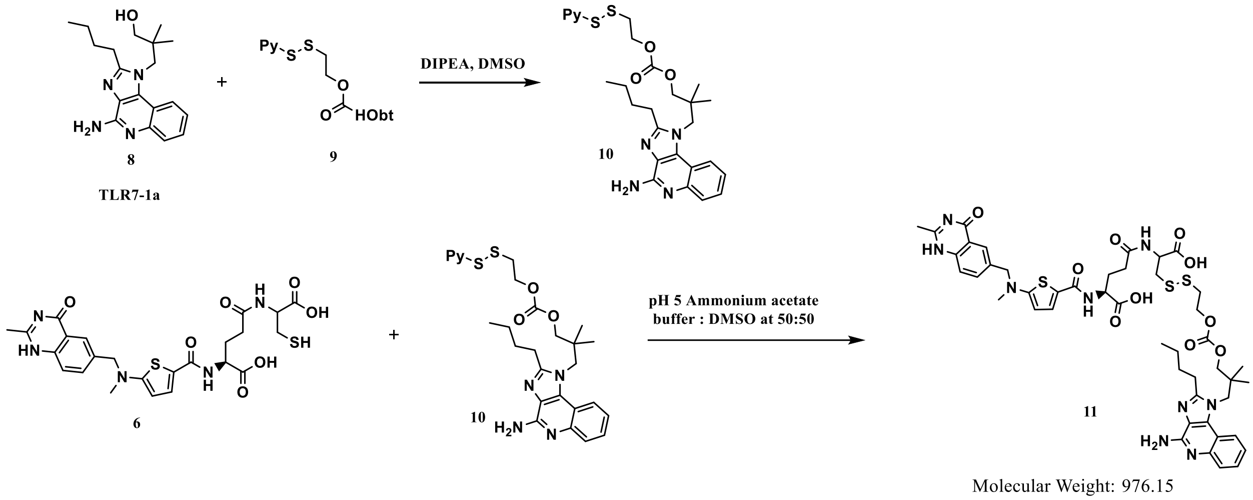

Supplemental Figure 7. Synthetic scheme for the preparation of raltitrexed-TLR7-1a (11) conjugate.

Synthesis of compound 10: To a solution of TLR7-1A (0.1 mmol) in DMSO, DIPEA (100 µl) and 1H-benzo[d][1,2,3]triazol-1-yl (2-(pyridin-2-yldisulfaneyl)ethyl) carbonate **9** (0.3 mmol) were added and stirred for 12 h. The crude reaction mixture was then purified by HPLC using 5-95% water in acetonitrile mobile phase, and the combined purified fractions were lyophilized to yield product **10** as a colorless crystalline solid (Yield ~33%).

Synthesis of compound 11: To a solution of raltitrexed-cysteine compound **6** (0.1 mmol) in 1:1 mixture of aqueous ammonium acetate (pH -5) and DMF, compound **10** (0.3 mmol) was added and stirred for 12 h. After the starting material was completely consumed, the crude reaction mixture was purified by UHPLC using 5-95% water in acetonitrile mobile phase. The combined purified fractions were lyophilized to yield raltitrexed-TLR7-1A conjugate (**11**) as a colorless solid (yield ~63%). The product is confirmed by LCMS (M.Wt. 976.15g/mol).

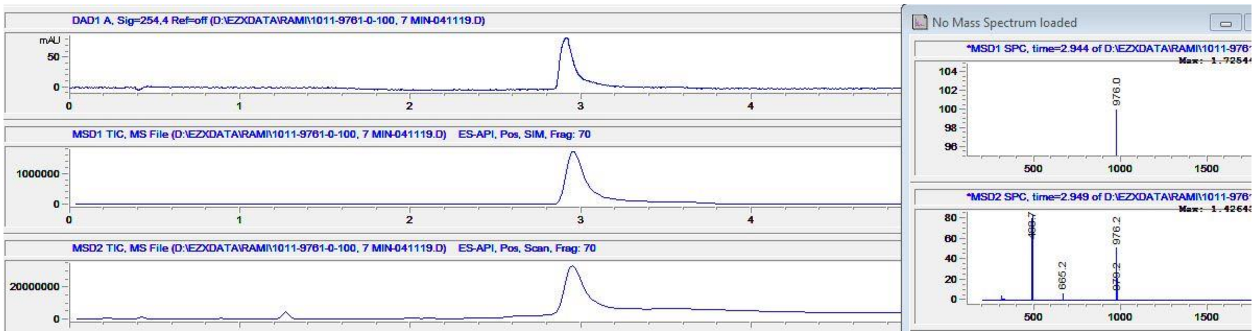

Supplemental Figure 8

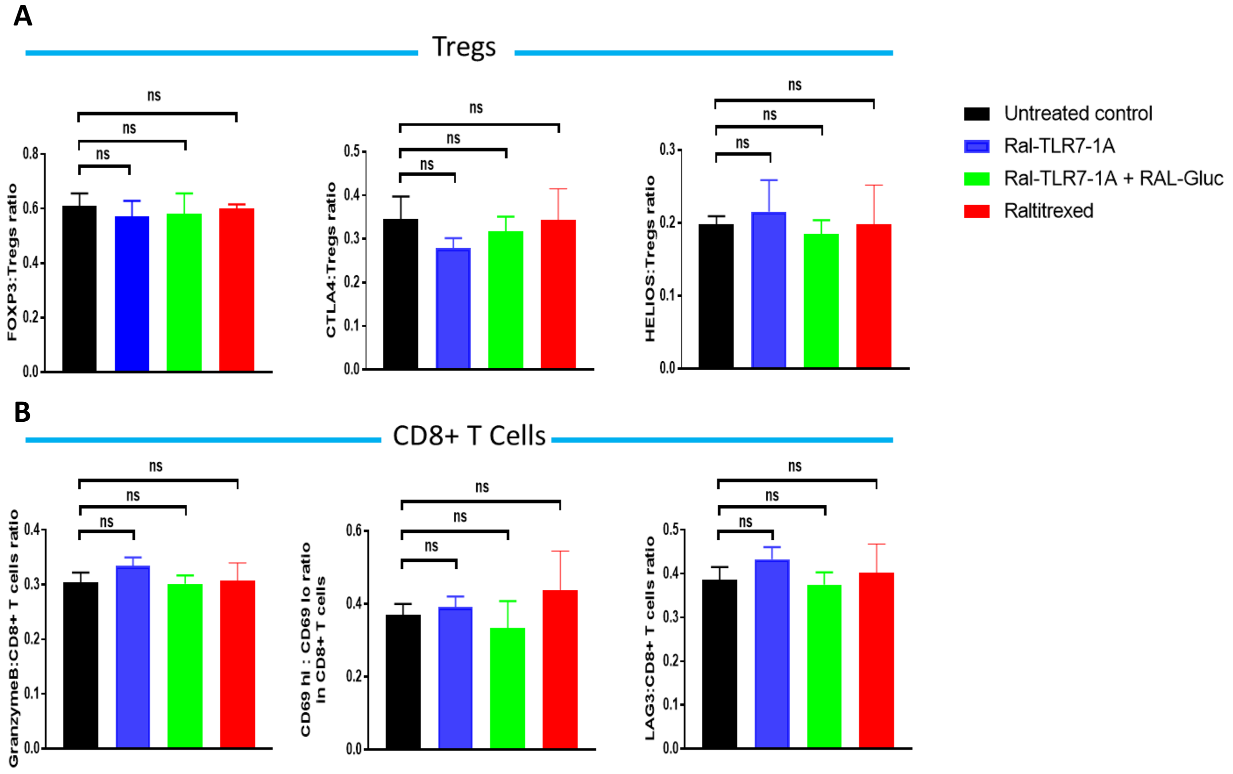

Supplemental Figure 8. Evaluation of the effect of Ral-TLR7-1a on phenotypic markers of splenic Tregs (A) and CD8+ T cells (B) from the tumor-bearing mice in Fig. 4A. Following euthanasia, spleens were excised and dissociated using collagenase prior to analysis of the component cells by flow cytometry. The relevant phenotypic markers are listed on the y axis and the treatment regimen is indicated by the color of the bar. (n=5 mice/group, mean ± SEM; ns, $P > 0.05$).

Supplemental Figure 9

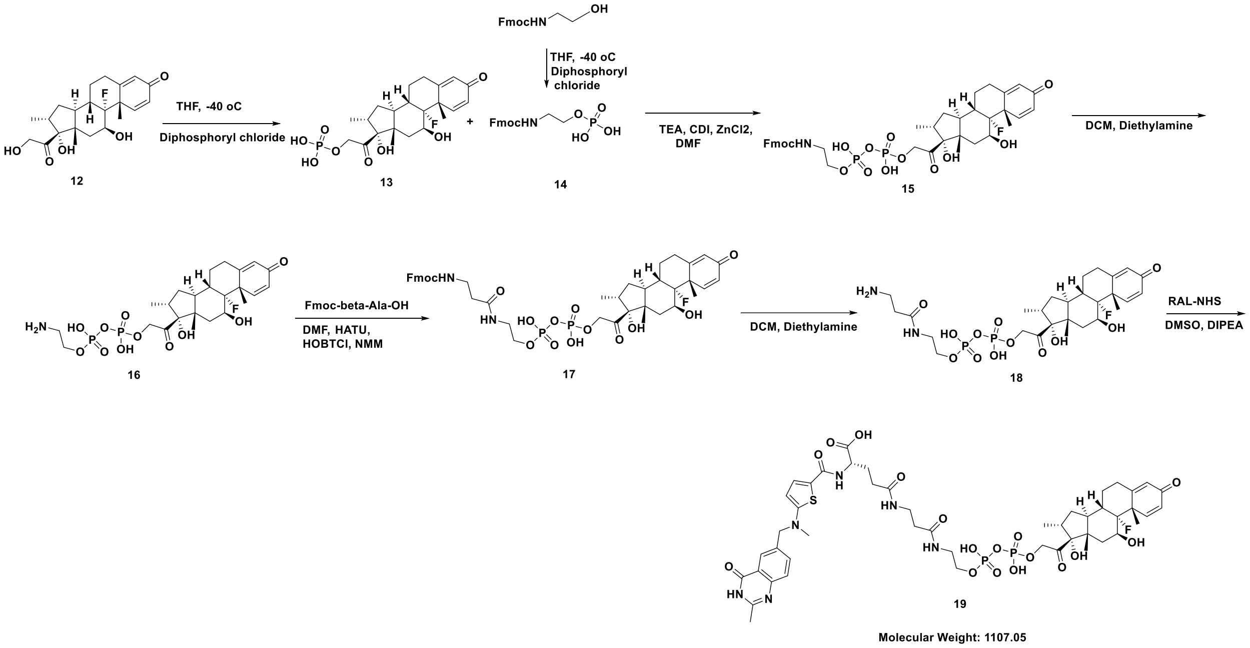

Supplemental Figure 9. Synthetic scheme for the preparation of raltitrexed-dexamethasone (19) conjugate.

Synthesis of compound 13: Dexamethasone (500 mg) was added to a 50 mL round bottom flask followed by anhydrous THF (2.5 mL). Then, the reaction was cooled to -40 °C with a dry ice /acetonitrile cold bath. 0.53 mL (3 eq) of diphosphoryl chloride was added and stirred at -40 °C for 1 h. The reaction was quenched with water and titrated to pH˜ 8 with saturated aqueous sodium bicarbonate solution. The solution was made acidic pH˜ 2 using a 1 N HCl solution and extracted several times with ethyl acetate. The combined organic phase was dried over anhydrous Na_2SO_4 then removed under the vacuum and used in the next step without any further purification (yield = 0.51 g).

Synthesis of compound 15: (9H-fluoren-9-yl)methyl(2-Hydroxyethyl)carbamate (500 mg) was added to a 50 mL round bottom flask followed by 3.4 mL anhydrous THF. Then, the reaction was cooled to -40 °C with a dry ice /acetonitrile cold bath. 0.6 mL of diphosphoryl chloride was added and stirred at -40 °C for 1 h. The reaction was quenched with water and titrated to pH˜ 8 with saturated aqueous sodium bicarbonate solution. The solution was made acidic pH˜ 2 using a 1 N HCl solution and extracted several times with ethyl acetate. The combined organic phase was dried over anhydrous Na_2SO_4 then de vacuo and used in the next step without any further purification (yield = 0.64 g).

To a stirred solution of 395 mg of Fmoc-phosphate in DMF (3 mL) were added triethylamine (0.16 mL) and CDI (0.45 g). The resulting solution was stirred at room temperature for 30 min. To this

mixture were added Dexamethasone21-phosphate (500 mg) and ZnCl2 (1.18 g), and the mixture was allowed to stir at room temperature overnight. The reaction was diluted with 1 N HCl and extracted several times with ethyl acetate. The combined organic layers were concentrated, and reverse phase column chromatography using combi flash (yield = 255 mg).

<u>Synthesis of compound 16:</u> To a stirred solution of Fmoc-pyro-Dex (255 mg) in DCM (4 mL) was added diethylamine (1 mL) and the resulting mixture was stirred at room temperature for 2 h. The DCM/DEA mixture was removed under vacuum (yield = 185 mg).

<u>Synthesis of compound 17:</u> A solution of 95 mg of Fmoc-β-Ala-acid was activated with 85 mg of HATU, 85 mg of HOBTCl, 11 mL DMF and 0.3 mL NMM. This mixture was then added to Fmoc deprotected pyro-Dex residue (185 mg) and stirred for 3 h for completion of the reaction monitored by LCMS. The reaction mixture was purified by reverse phase column chromatography using combi flash (yield = 120 mg).

<u>Synthesis of compound 18:</u> To a stirred solution of FmocNH- β-Ala-pyro-Dex (120 mg) in DCM (4 mL) was added diethylamine (1 mL) and the resulting mixture was stirred at room temperature for 2 h. The DCM/DEA mixture was removed under vacuum (yield = 80 mg).

<u>Synthesis of compound 19:</u> Raltitrexed (100 mg), N-hydroxyl-succinimide (31.3 mg), and dicyclohexylcarbodii- mide (46.7 mg) were dissolved in dry DMSO (10 mL). The reaction mixture was stirred, in the dark, at room temperature for 15 h. The NHS-raltitrexed was precipitated by the addition of ethyl acetate, filtered, and washed with ethyl acetate once and anhydrous diethyl ether thrice. The NHS-raltitrexed was dried under a desiccator and used immediately. Fmoc-deprotected compound (15 mg) was dissolved in DMSO (1 mL) then added NHS-raltitrexed and DIPEA. The reaction was monitored by LCMS and upon completion was purified by RP-HPLC and verified by LCMS (yield = 3.5 mg).

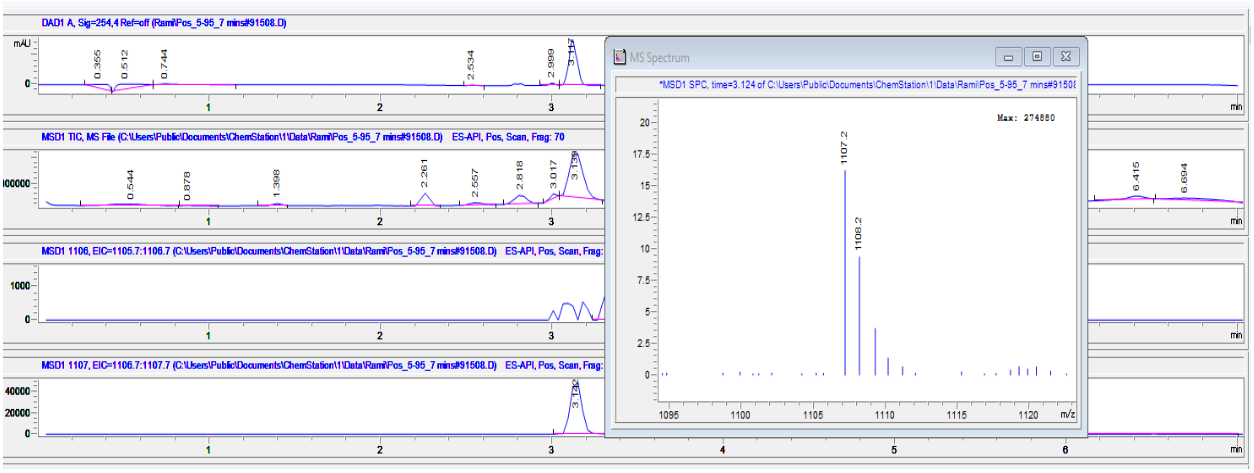

Supplement: Supplementary file 1 [file DataSheet_1.pdf]
